# The Sanford Lorraine Cross Award for medical innovation: Advancing a rigorous and repeatable method for recognizing translational research leaders who today are bringing emerging transformative innovations to patients

**Published:** 2020-01-28

**Authors:** Mitchell Horowitz, Joseph Simkins, David A. Pearce

**Affiliations:** ^1^TEConomy Partners, LLC, Bethesda, Maryland, United States of America; ^2^TEConomy Partners, LLC, Dublin, Ohio, United States of America; ^3^Sanford Health, Sioux Falls, South Dakota, United States of America; ^4^Pediatrics and Rare Diseases Group, Sanford Research, Sioux Falls, South Dakota, United States of America; ^5^Department of Pediatrics, Sanford School of Medicine, University of South Dakota, Sioux Falls, South Dakota, United States of America

**Keywords:** research awards, medical innovation, translational research

## Abstract

Medical innovation awards stand out as an important means to focus public attention on what matters in medical advancement. Traditional awards typically focus on celebrating medical innovators with either a track record of proven successes in new treatments or promising basic science breakthroughs still years away from reaching patients. Perhaps one of the greatest challenges for medical innovation that is not sufficiently addressed by these traditional awards is celebrating translational research efforts on the cusp of major advancements where medical innovators are demonstrating success in bringing emerging transformative medical innovations to patients. As a part of its award process, the Sanford Lorraine Cross Award has developed a unique method to fill this gap in the landscape of medical innovation awards for ongoing translational research efforts by identifying promising medical innovations within a narrow spectrum of the research pipeline on the verge of having transformative impact for patients in the near term. The Sanford Lorraine Cross Award addresses the challenges of identifying emerging transformative medical innovations making their way through development by deploying a rigorous, analytically-based “early signals analysis” to identify emerging transformative medical innovations in its selection process independent of the medical innovators who are succeeding in bringing them forward. It also stands apart from traditional medical innovation awards in focusing on identifying award candidates that have significant roles in bringing the emerging transformative medical innovation across the finish line to patients, and their efforts in overcoming challenges, forging collaborations, and ensuring a successful outcome. The data-driven award selection process used for the Lorraine Cross award ultimately inverts the standard medical award selection paradigm – truly innovative areas of discovery and breakthrough science are identified independently of candidates and used to then focus candidate selection on the areas with the most promising transformative potential for patients. This article sets out the details of how the Sanford Award makes use of leading tools and methods in identifying transformative innovations currently in translational research to provide another important focus of what matters in medical innovation.

**Relevance for Patients:**

The Sanford Lorraine Cross Award identifies the most successful application of translational research that ultimately expedited the development of a treatment or cure of a disease.

## 1. Introduction

Medical research awards garner significant attention and prestige in recognizing research excellence and contributions to making a difference in advancing medical innovation. For instance, the NIH Almanac touts that “The National Institutes of Health (NIH) has a long, rich tradition of support for award-winning, cutting-edge research. Many of the world’s most distinguished investigators have been honored with medicine’s top prizes, including the Nobel Prize and awards from the Albert and Mary Lasker Foundation – ‘America’s Nobels’ – honoring groundbreaking contributions to our understanding of the human disease” [1]. This past September, the Journal of the American Medical Association featured a section on the recipients of the 2019 Lasker Awards [2].

These traditional medical innovation awards either celebrate those medical researchers and innovators with proven successes or transformative basic science breakthroughs still years away from reaching patients. Among the examples of major awards for proven successes are the Nobel Prize in Physiology or Medicine with an emphasis on “discoveries that have changed the scientific paradigm and are of great benefit for mankind” [3] and the Lasker Award recognizing “contributions of scientists, physicians, and public servants who have made major advances in the understanding, diagnosis, treatment, and prevention of human disease” [4]. On the other extreme, recent basic science advances that have not yet advanced to reach patients are the Breakthrough Prize in Life Sciences focused on “transformative advances toward understanding living systems and extending human life” [5].

While these traditional medical research awards are a critical component of celebrating foundational scientific research, there is not currently a focus on celebrating those medical researchers and innovators closing the translational research gap for emerging transformative medical innovations today as opposed to the past or in a distant future. Advancing current translational research is perhaps the most pressing challenge of today’s age in medical research in terms of providing new health-care solutions to patient populations. As the groundbreaking Food and Drug Administration (FDA) report on the Challenges and Opportunity on the Critical Path to New Medical Products (commonly referred to as the Critical Path Report) brought to public attention that “at a time when basic biomedical knowledge is increasing exponentially, the gap between bench discovery and bedside application appears to be expanding” [6].

The need to recognize and incentivize medical researchers and innovators having success in addressing today’s translational research challenges calls for a new type of medical innovation award backed by an analytical process that leverages the holistic body of highly descriptive but unstructured data on medical research activities. The importance of translational research requires a new paradigm that incorporates means to assess the emerging transformative medical innovations that are making their way through the development process to reach patients. A medical innovation award process that embraces the context of translational research also needs to be able to identify the medical researchers and innovators making significant contributions in advancing these emerging transformative medical innovations by demonstrating ingenuity, perseverance, and commitment to its success in reaching patients.

Developing a rigorous and repeatable method for creating an award for translational research is the vision and focus of the Sanford Lorraine Cross Award, which celebrated its inaugural award in emerging transformative medical innovations in December 2018 and is now preparing for its second award process for December 2020.

**Figure 1:**
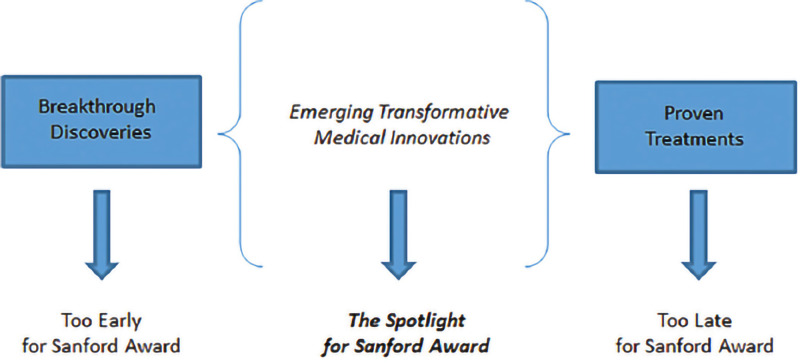
The unique award focus of the Sanford Lorraine Cross Award.

This award is agnostic to individuals at outset. As depicted in Figure 1, the goal of the award is to identify promising medical innovations within a narrow spectrum of the research pipeline on the cusp of having transformative impact for patients and can benefit from concerted research support to reach significant treatment milestones in the near term.

Another unique aspect of the Sanford Lorraine Cross Award is the criteria used to select the award winners. Rather than primarily focusing on the significance of the contribution of a researcher or clinician, the Sanford Lorraine Cross Award is concerned about the role that the award candidate has played in bringing a new emerging transformative medical innovation across the finish line to patients, and their efforts in overcoming challenges, forging collaborations, and ensuring a successful outcome.

A final distinguishing aspect of the Sanford Lorrain Cross Award is the “rigor” it brings in focusing on emerging transformative medical innovations in its selection process independently of the pioneers who are succeeding in bringing them forward. In particular, the Sanford Lorraine Cross Award stands out in the development of an “early signals analysis,” which applies a data-driven approach using advanced analytical techniques to capture the vision of the Sanford Lorraine Cross Award in targeting emerging transformative medical innovations. As explained in further detail below, the early signals analysis relies on machine learning approaches to enable Sanford to cast a wide net across an expansive portfolio of high-impact translational research activity indicators and identify those innovations that are on the cusp of realizing transformative impacts in bringing new treatments to patients.

## 2. Methods

**Figure 2:**
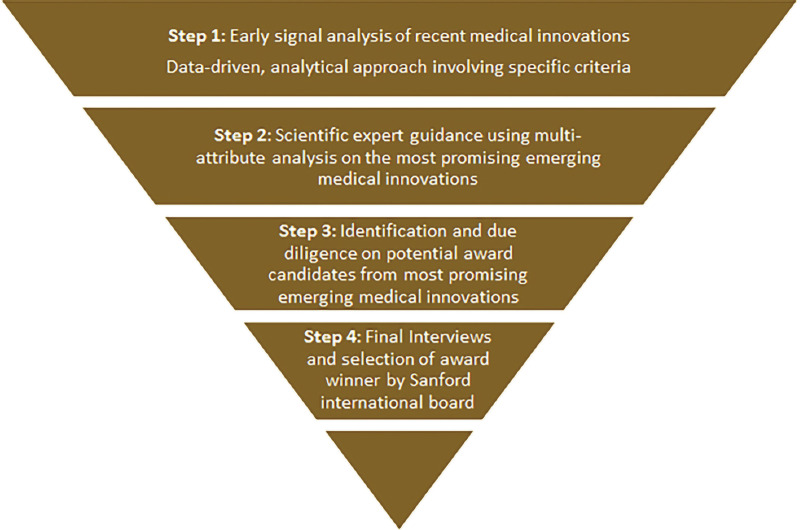
Four-step process to Sanford Lorraine Cross Award.

The early signal analysis functions as the first step in a four-step selection process for the Sanford Lorraine Cross Award, as depicted in Figure 2. Once identified by the early signal analysis, the leading medical innovation areas were further validated and refined by a panel of scientific experts to help systemically evaluate the “transformative value” of the innovations. A multi-attribute survey approach that integrated the assessment by the scientific experts of each medical innovation area in respect to its significance in advancing the state of medical science, improving clinical practice, driving significant impacts on patient health, and addressing broader public health issues was used to help assess medical innovation areas in an unbiased review process that considered the full scope of potential transformative impacts. A multi-day review session was convened with the scientific experts, who used the multi-attribute assessment as a starting point for their discussions and guidance on which emerging medical innovations stood out as transformative and poised to make a difference in patient’s lives.

Following the selection of the most promising emerging transformative medical innovation areas, the focus then shifted to identifying specific candidates who are advancing science in those areas and embody the spirit of the Sanford Lorraine Cross Award. Potential candidates were identified based on peer-reviewed sources detailing the research history and current status of those selected emerging transformative medical innovation areas, and data mining of recent online activity in medical innovation news and press releases related to those areas. Further, due diligence was carried out by Sanford Health, including discussions and in-depth background analysis of candidates to identify the top three candidates for consideration.

The ultimate selection among the three final candidates, each of which was well-qualified, is the responsibility of the Sanford International Board to ensure the focus on a medical pioneer who has demonstrated ingenuity, perseverance, and commitment to bringing an emerging transformative medical innovation to fruition. The Sanford International Board offers both a strong patient-orientation along with a passion for supporting the improvement of the human condition through transformative medical treatments and care, and brings their expertise and life experiences as entrepreneurs, health-care leaders, business executives, and world-class competitive athletes to bear. This advisory group helps oversee Sanford’s World Clinics, with current locations in Canada, China, Germany, Ghana, and the U.S. These Sanford World Clinics provide care to children, families, and underserved populations along with offering innovative approaches in areas as primary care services, regenerative medicine and diabetes, to improve the health and well-being tailored to the needs of each community it serves, using methods that surpass current practices of health-care delivery.

### 2.1. Early signal analysis approach

This rigorous early signals analysis stands in contrast to other major medical innovation awards, which generally rely on the subjective vetting of a small set of highly accomplished and recognized scientific leaders (Table 1) and reflects the goal of bringing forward unheralded pioneers and supporting their efforts in reaching patients.

**Table 1:** Nominating and selection process of major medical innovation awards.

Other major medical innovation awards use nominating processes and review committees and panels to select candidates
• Nobel Prize in Physiology or Medicine invites over 3,000 persons who hold positions suggesting they are competent and qualified to nominate candidates each year, with self-nominations not considered and the list of nominees not made public for 50 years. The nominees are then reviewed by a committee of six comprised five members elected from the 50 member Nobel Assembly and the secretary of the Nobel Assembly, which can solicit evaluation reports from experts to prepare evaluation reports of the nominated candidates. Recommendations are made by the committee to the full Nobel Assembly, which votes to select the award winner [3]
• Lasker awards have an open, online nominating process and then uses different juries of experts to select from the nominees for its different awards [4]
• Breakthrough Prize in Life Sciences uses an open, online nominating process with self-selection not permitted. Past recipients of the prizes are invited to serve on the Selection Committee to select recipients of future prizes [5]

**Figure 3:**
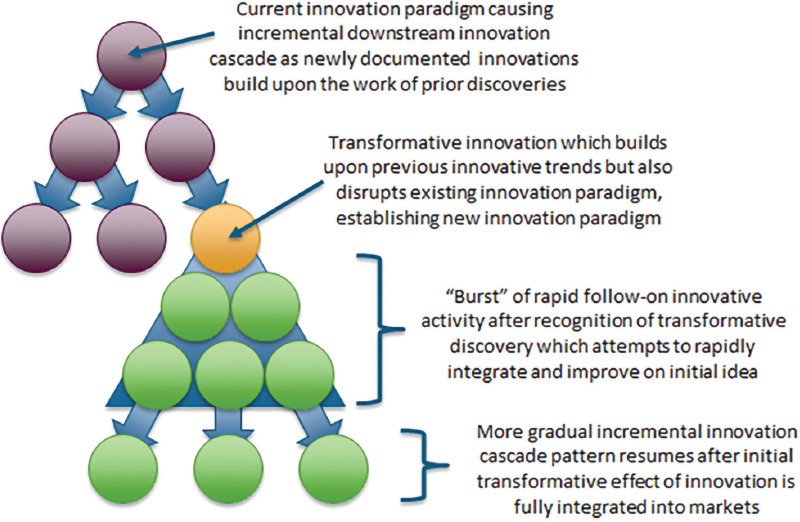
Cascading patterns of innovation and “bursts” in activity after introduction of disruptive ideas.

The approach of using early signals to identify trends in innovation is rooted in the scientific literature of how innovations evolve over time and the “bursts” in innovative activity that typify emerging innovations. One way of describing trends in innovation is using the analogy of a cascade, shown visually in Figure 3, where initial innovative activity seeds ideas for downstream innovations to then generate subsequent innovations in a cyclical pattern. In their work on identifying transformative scientific research, Huang *et al*. explained that when disruptive innovation occurs in this cascade of innovative development, it disrupts the existing paradigm and begins a new innovative cascade of ideas generated by the disruptive idea [7]. These disruptive ideas are indicative of the types of innovative leaps the selection process seeks to identify among the vast body of ongoing research and scientific discovery activities occurring at any given point in time.

Analyses of this type of disruption rely on the linkages between ideas, and for this reason, the forward citation patterns, or citing of past work by future work, of research publications, and patents are often used to study shifts in patterns that indicate the onset of a disruptive idea. Other work on identifying and characterizing high-impact and transformative science metrics also relies on the idea that transformative ideas will rapidly generate various types of measurable recognition and forward citation activity as radical and high-impact ideas disrupt previously established citation patterns and generate new concepts [5].

The concept of using the patterns related to short-term bursts of innovation activity that occurs just after a transformative or disruptive idea is introduced to characterize high impact areas through forward citation analysis is reflected in various ways using several different approaches and metrics through the work of a number of other researchers, and is particularly applicable to the biomedical innovation space [8-10].

### 2.2. Identification of early signal measures

Since the Sanford Lorraine Cross Award is intended to recognize emerging transformative medical innovations making their way through development with a clear path to reaching patients, only signals that occur at the later stages of basic research up until just before product introduction to market are included to spotlight developing areas where the award can help propel emerging ideas to completion.

The identification of early signals consistent with the focus of the Sanford Lorraine Cross Award requires tracking activities that take place across translational research. Translational research is the pathway in which basic research discoveries are advanced and developed into new innovative medical products to serve patients, and it reflects the critical interface of “bench and bedside” relationships which drives medical innovations forward. The U.S. NIH explains that: “Information flow at this interface is bi-directional, requiring close interaction between clinical and bench scientists” [11]. Translational research is a complex continuum across which industry-academic collaborations occurs with a high degree of bi-directional interaction between basic, applied, and clinical sciences. A 2015 study by the Tufts Center for the Study of Drug Development found that nearly 80% of the most transformative new drug innovations over the past 25 years resulted from collaborations between industry and academic research [12].

The nature of translational research and the many steps involved in advancing bioscience innovation makes it difficult to establish a single metric intrinsically tied to its development. The early signals data sources considered in an analysis of innovative potential should represent a variety of milestones on the timeline of translational research as medical innovations go from initial discovery to market delivery to ensure maximum coverage, as transformative applications can occur at various points in the process of developing an initial breakthrough discovery into a market-ready product.

For this reason, a mix of seven early signals was identified for the Sanford Lorraine Cross Award to consider, including:

- NIH transformative research grants
- High impact scholarly activities
- High impact patent activities
- High potential venture-backed companies
- High potential NIH small business innovation research grants
- FDA expedited review
- Trending social media topics in medical innovation

Table 2 explains the rationale for the inclusion of each early signal and its limitations.

**Table 2:** Sources of early signals data documenting potentially transformative medical innovations.

Early Signals Data Source 2	Rationale for Inclusion	Limitations	Current Data Sources
Trends in Online News/Announcement Activity	Can potentially identify brand new innovations that have not appeared in any of the other more formal signal metrics and do not rely on any review or publishing process that might induce bias toward certain types of innovative concepts	No way to attach measures of risk or importance to innovations that appear in these signals, so very high uncertainty in the eventual success of innovations described unless corroborated by other early signals areas	Web Scraping of Research/Innovation-Related Posts from Selected Websites

High Impact Research Publications	Publication in peer-reviewed sources shows legitimacy to value of new research in medical fields that can identify promising discoveries in advance of the commercialization process	Potential bias exists in journal publications towards less risky/radical innovations that do not deviate significantly from the current scientific consensus	Thomson Reuters/Clarivate Analytics Web of Science Research Publications Database

NIH Transformative Research Awards	Can capture promising innovative concepts in their earlier stages of testing and validation that are backed by leading academic and clinical researchers	Not all promising medical technologies will rely on grant award funding, so this signal may be biased toward current NIH research agenda and award trends	NIH RePORT Database

High Impact Patents	Can serve as one of the first public-facing signals of promising developments in new technology areas, with detailed technology profiles available documenting innovative development	Dynamics of patenting trends sometimes make it difficult to definitively identify specific patents which had a transformative impact until many years later	USPTO Database via Thomson Innovation/Clarivate Analytics Innovation

SBIR Awards	Can identify very early stage companies with potentially transformative technologies just after the proof of concept stage with some indication of the commercialization validity of initial ideas	Often is not possible to distinguish the impact of innovations developed through SBIR awards	US Small Business Administration SBIR/STTR Awards Database

Early Stage Venture Capital Investment	Early stage backing by private venture capital sources can serve as an indicator that startups are based around technologies that are perceived to have high potential for market applications	The significant startup failure rate is usually built into investment decisions at early stages so less certainty around the eventual success of candidates	Thomson One Venture Capital Investment Database

FDA Expedited Review Programs	Special designation by FDA expert reviewers is a clear indicator of the transformative potential of medical innovations	Can only spotlight innovations just before or at approval for use on the market, meaning metric is biased more toward proven technologies and does not capture riskier upstream research efforts	US FDA CDER and CDRH Annual Reports

RePORT: Research Portfolio Online Reporting Tools, USPTO: US Patent and Trademark Office, CDER: Center for Drug Evaluation and Research, CDRH: Center for Devices and Radiological Health

### 2.3. Approach to measuring and defining the content of the early signals

From the scientific literature, a critical way to identify those high impact innovations reflecting a “burst” of activity is through various measures of forward citation patterns. To the extent possible, this focus on forward citations patterns as an early signal of high volume, compressed innovation activity is used to process signals data (such as for publications and patents). However, this is not possible for all measures due to limitations in the underlying data. When forward citations are not available for identifying high potential innovation activities taking place in specific early signal measures, then the early signal measure itself is defined in a manner that reflects only highly innovative activities, such as only selecting innovations going through clinical trials approved for expedited reviews.

Table 3 shows the specific criteria used for each of the early signal areas. Where forward citation information is available, such as research publications and patents, citations data detailing the downstream activity linked to innovations over the past 5 years were used to identify records in each year, which were outliers in terms of high forward citations activity that had especially high potential to be involved in bursts of innovation. Where citation information was not available, the early signals data included were based on selection criteria that targeted characteristics or programs that indicate a particular focus on transformative impacts or emphasize translational research outcomes demonstrating progress toward a path to market.

**Table 3:** Key selection criteria used to filter early signals data sources for innovations with high transformative potential.

Early signals data source	Key selection criteria
Trends in Online News/Announcement Activity	• Include only web surveillance on innovation from set of biomedical-focused science and technology blogs and social media accounts with track record of recognizing innovative discoveries as selected by Sanford and other medical experts

High Impact Research Publications	• Include articles from key journal set that has record of publishing research related to medical innovation, as identified by journal index measures • Identify articles in key journals over past 5 years which have within-journal, within-year forward citation levels three standard deviations or higher above-average levels

NIH Transformative Research Awards	• Only include research project awards (R01 equivalents, cooperative agreements, and other project grants) over the past 5 years • Only consider grants funded through Office of the Director/Office of Strategic Coordination, which is key source for NIH transformative research awards, NIH Common Fund research areas (cross-center collaborative research in high priority areas), and other high priority/expedited research funding sources

High Impact Patents	• Identify provisional patent applications over the past 5 years in patent classes that are focused around biomedical technologies using detailed patent class definitions related to diagnostic, therapeutic, and medical device applications • Identify patents which have within-detailed patent class, within-year forward citation levels three standard deviations or higher above-average levels

SBIR Awards	• Include only Phase 2 awards that demonstrate progress on validating concepts which received initial Phase 1 awards • Only consider awards from NIH in major disease areas to limit the scope of company applications to potentially transformative biomedical areas

Early Stage Venture Capital Investment	• Only consider companies in biomedical and biotech industry classifications • Identify companies receiving greater than $10M in funding in early rounds (seed/early stage) over the past 5 years as an indicator of high transformative potential

FDA Expedited Review Programs	• Approvals data from key fast track and innovation programs at CDRH and CDER over the past 5 years: • CDER New Molecular Entity/New Therapeutic Biologic Designation • CDER Breakthrough Therapy Designation • CDER Accelerated Approval Designation • CDRH Device Pre-Market Approval Expedited Review

**Figure 4:**
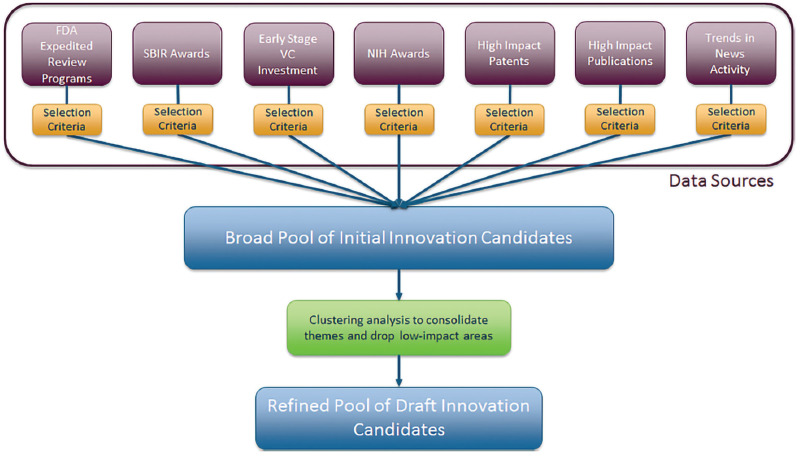
Selection process for initial pool of candidate transformative medical innovations.

Once filtered, the unstructured text content present in the early signals data describing the applications of each innovation is pooled for further analysis of the innovative technology platform themes present across the breadth of the various early signals measures. This unstructured text data takes the form of descriptive metadata attached to each record documenting an innovation and includes information such as patent and publication abstracts, grant award descriptions, early stage company descriptions, and social media posts describing innovative discoveries. The various early signals records are pooled without any weighting attached to individual records since later steps in the early signals validation process incorporate subject matter expert judgment and feedback in weighting the validity of different concepts. The complete process of selecting, filtering, and combining the early signals data for use in subsequent analysis phases (Figure 4).

### 2.4. Methodology for early signals clustering and validation analysis

There are a wide variety of different quantitative approaches that are possible for analyzing the thematic makeup of early signals data, each of them with advantages and limitations. A comprehensive review by Cozzens *et al*. of quantitative methodologies for identifying emerging technologies indicates that the two most commonly used types of analysis for characterizing the information structure of data rely on patterns formed by either keywords or citations [13]. The methodology used here for identifying potentially transformative innovations through a signals analysis approach represents a hybrid of these two approaches that attempts to first narrow the field of potential innovations to those that display high potential for generating transformative innovation and then categorizes the descriptive text content of the records documenting those innovations into innovation theme areas that can be evaluated by scientific experts to determine their value.

**Figure 5:**
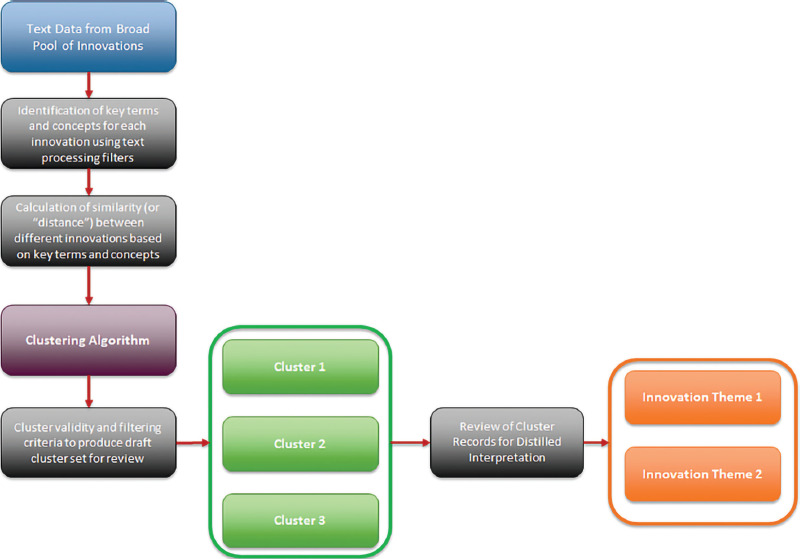
Process for thematic clustering analysis of refined pool of medical innovations from early signals data.

To analyze the overarching themes present in the refined pool of early signals innovation data, a machine learning technique known as unsupervised latent topic modeling analysis is used to build out “vocabularies” based on the unstructured text content and then use them to identify distinct topics present across the data present in the descriptions. A text processing algorithm is used to clean the text data and then identify frequently appearing terms and multiword phrases through techniques such as word stemming and stop-word removal which are commonly used in natural language processing methods, which turn text content into data for analysis. Once key terms are identified for each record, weighted term frequency methods are used to assess the importance of each term or keyword phrase in describing the content held within an individual text record. The key terms identified for each body of unstructured text are then used as inputs to an unsupervised clustering technique known as latent topic modeling to identify key underlying concepts present in the text data based on the process of building out “vocabularies” of terms that are identified by the algorithm and evaluating their presence across the data. As opposed to more basic clustering algorithms which evaluate text content at the overall record level and then assign a unique theme to a data record, latent topic modeling estimates the mixture of topics present in an individual record to better approximate the structure of real-life text content which often contains multiple themes within a single record. In addition to identifying the underlying topic structure present across the body of text data, the algorithm also enables calculation of a measure of similarity, or “distance,” between different text records documenting innovations so that all records in the refined pool of early signals data can be compared to one another to identify records that contain similar ideas and concepts. The steps for processing the early signals data for the next stage of review by scientific expert panels are shown in Figure 5.

Even after identification of an initial set of topic clusters by the clustering algorithm, it is still necessary to evaluate the cluster groupings to determine cohesiveness and critical mass around a single relevant subject based on analysis of key terms appearing in cluster records. These validation steps filter out clusters that are not focused or are not relevant to the innovation areas that the Sanford Lorraine Cross Award process is designed to target.

A total of 20,290 records were generated from the sources for the early signal analysis. This included 9218 records from standardized databases that document more formal stages of innovation involving: Publications, NIH grants, patents, early stage venture capital, SBIR Phase 2 awards, and FDA special approvals. Another 11,072 records came from web surveillance data across medical innovation and research news aggregators that offer insights on informally recognized areas of medical innovation that has new and exciting developments.

The latent topic model identified approximately 100 underlying topics present in the body of early signals data. These initial topics were then interpreted through expert review validation and categorized as relevant or “artifact” topics based on how highly focused their themes were on biomedical innovation topics versus other subjects. This step identified 72 highly focused biomedical innovation topics for review by Sanford stakeholders to assess their relevance to the specific vision of the Lorraine Cross Award with respect to the maturity and ongoing research activity in the innovation area. From this grouping, a total of 57 medical innovation topics from the text records were identified and then underwent subsequent review steps with a group of internal Sanford scientific experts who identified a final set of 14 medical innovations to consider in the validation exercise with the external scientific expert panel.

### 2.5. Engaging scientific experts to finalize the identification of emerging transformative medical innovations

In selecting among potential emerging medical innovations for those that stand out as having the highest transformative potential, there is no substitute for the inclusion of expert judgment in evaluating the transformative potential of medical innovation. As Huang *et al*. note in their work outlining processes by which transformative research can be identified that there are many cultural and cognitive biases that can need to be considered. Further complicating the identification of medical innovations is a wide range of different attributes to innovations that must be considered and weighed, such as potential for impacts on scientific discovery, patients, clinical practice, and public health.

There is an existing literature on retrospective analyses of transformative innovations in the medical space, which includes a variety of techniques that utilize subject matter experts to either critique or rank order sets of medical innovations based on present-day recognition of the innovative value of a set of innovations. For example, Kesselheim *et al*. [14] use a method of repeated surveys (known as a Delphi survey protocol) of a large group of physicians to distill a listing of the top transformative drugs of the past 25 years, while Fuchs and Sox [15] ask a group of physicians to rank a group of 30 major medical innovations using their implied relative importance to treatment of patients. Both of these examples highlight the importance of accurately capturing tradeoffs in key benefits of medical innovations across a variety of potential disease areas and applications as well as utilizing methods to account for the biases of respondents in reaching a consensus among expert respondents on the transformative value of different innovations.

For the Sanford Lorraine Cross Award, the difference from retrospective studies is providing a rigorous approach to conducting the due diligence on emerging innovations identified through the early signals analysis that accounts for the role of uncertainty and the implied preferences of respondents in a forward-looking way. One way to do this is to use a decision theory framework known as multi-attribute utility modeling to capture expert opinions across the various defining characteristics of a particular innovation. Utility modeling techniques are used to assign preference rankings to choices across a set of uncertain outcomes, in this case, represented by the uncertain outcome of the ultimate future transformative impact for a group of candidate medical innovation applications areas.

Multi-attribute modeling relies on two key pieces of information gathered from a survey process and a utility function to determine the “utility” of each medical innovation in the eyes of scientific expert evaluators with respect to its ability to cause transformative impacts. The information provided by expert survey respondents is comprised attributes or descriptive properties or characteristics of medical innovations that are measured or scored, and weights, or the importance of each attribute to the respondent in determining the transformative potential of a medical innovation. The combination of attribute scores and weights using a utility function produces a measure of the transformative potential of a given medical innovation, which can then be used to comparatively rank medical innovations on a consistent basis to determine the top candidate innovation areas to consider in identifying candidate inventors for award consideration. For this analysis, the utility function used to combine attributes and weights takes the form of a simple weighted average:


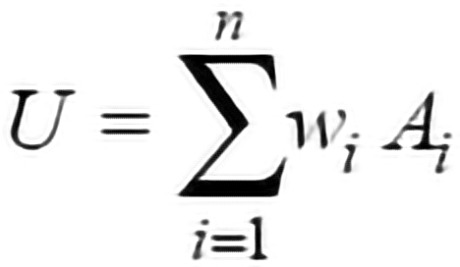


where

*U* = the overall utility measure for a particular candidate medical innovation

*n* = the number of attributes used to describe medical innovations

*w* = the importance weights of different attributes

*A* = the scores for different attributes

While other utility function forms are possible, this particular form of multi-attribute analysis is commonly used across a variety of technology areas to assess the market potential for products and services.

A review of the literature describing the profiles of the past transformative medical innovations was used to outline a set of attributes describing a set of characteristics of transformative medical innovations. Attributes were identified within the context of four broad areas that describe the transformative impact of innovations on applications of medical science and refined with guidance from a scientific expert group composed of Sanford researchers and clinicians to validate their effectiveness in capturing the dimensions of what it means to have transformative effects. The final listing of broad impact areas that contain detailed attributes across which survey respondents were asked to evaluate candidate medical innovations is shown in Table 4.

**Table 4:** **** Attributes of transformative medical innovations used to evaluate candidate innovation areas in the selection process.

Impact area	Attributes of transformative medical innovations
Scientific advancement	Displays novel mechanism of action or radically original approach to treatment

	Has the high potential to create downstream innovation and follow-on discoveries

	Displays a breadth of potential applications across multiple disease areas

Clinical practice	Allows clinicians to provide significantly improved diagnostic insights for patients

	Has significant potential to improve clinical efficiency or delivery of treatment

	Has significant potential to impact the practice field and treatment guidelines in one or more disease areas

Patient health	Displays improvement in treatment efficacy versus current practices

	Has significant potential to improve individual morbidity burden and patient quality of life or empower greater patient knowledge about their condition

	Has significant potential to reduce side effects or improve the safety of treatment

Public health	Has significant potential to impact a disease area with significant incidence rate and large affected population

	Has significant potential to reduce the indirect costs and burdens of disease on society

	Has a high potential impact on at-risk or underserved patient populations

	Has a high likelihood of being easily adopted and integrated into existing health care delivery systems

An accomplished and broad-based panel of scientific leaders was organized by Sanford Health to participate in the multi-attribute survey process and came together as a group to consider the results and to help guide the focus of what emerging medical innovation areas held the highest transformative potential from which to consider candidates. The members of the Sanford Lorraine Cross Scientific Advisory Board are shown in Table 5.

**Table 5:** Members of the Sanford Lorraine Cross Scientific Advisory Board.

**Mark A. Atkinson, Ph.D.,** University of Florida, Jeffrey Keene Family Professor, Departments of Pathology and Pediatrics; American Diabetes Association Eminent Scholar for Diabetes Research **Michelle L. Baack, M.D.,** Sanford Health, Boekelheide NICU, Neonatologist; University of South Dakota-Sanford School of Medicine, Department of Pediatrics, Division of Neonatology, Associate Professor of Pediatrics; Physician-Scientist, Environmental Influences on Health and Disease Group **Kym M. Boycott, M.D., Ph.D.,** Children’s Hospital of Eastern Ontario, Clinical Geneticist; CHEO Research Institute, Senior Scientist and Investigator; University of Ottawa, Department of Pediatrics, Associate Professor **Marilyn K. Glassberg Csete, M.D.,** University of Miami, Professor of Medicine, Surgery, and Pediatrics; Interstitial Lung Disease Program, Director; Pulmonary Diseases at Interdisciplinary Stem Cell Institute, Director; Vice-Chairman of Medicine for Diversity and Innovation **Deborah J. Fowell, Ph.D.,** University of Rochester Medical Center, Dean’s Professor of Microbiology and Immunology **Alison G. Freifeld, M.D.,** University of Nebraska Medical Center, Professor, Internal Medicine Division of Infectious Disease **William J. Pearce, Ph.D.,** Loma Lida University School of Medicine, Professor of Physiology; Center for Perinatal Biology, Associate Director Susan R. Rheingold, M.D., Professor of Clinical Pediatrics at the Perelman School of Medicine, University of Pennsylvania, Medical Director of the Outpatient Oncology Program, Children’s Hospital of Philadelphia **David A. Sinclair Ph.D.,** Harvard Medical School, Professor, Department of Genetics; Co-Director of Paul F. Green Center for Biological Mechanisms of Aging **Clive N. Svendsen, Ph.D.,** Cedars-Sinai, Kerry and Simone Vickar Family Foundation Distinguished Chair in Regenerative Medicine, Regenerative Medicine Institute; Professor in Medicine and Biomedical Sciences Joshua Wynne, M.D. MBA, MPH, University of North Dakota School of Medicine and Health Sciences, Dean, and Vice President for Health Affairs

Since transformative medical innovations identified in early signals data do not necessarily yet have a track record of creating impacts, the attribute evaluations relied on the expert judgment of transformative potential through relative scoring rather than measured outcomes criteria. A survey instrument was created and tested to validate this approach using the following process in two sequential steps:

- *Scientific experts were first asked to weight their preferences toward the attributes shown above with respect to their relative importance in determining the transformative potential of any new medical innovation in the marketplace. To accomplish this, a “budgeting” survey design was used to elicit implied preferences for the importance of certain areas through having respondents assign point values across the entire set of attributes shown above from a limited budget of total points. For this evaluation, a 100-point budget across the 13 attributes was used to determine importance weightings*.
- *Scientific experts were then asked to review a refined list of medical innovations identified from the early signals analysis using a Likert scale survey design to elicit measures for how they viewed the potential transformative impact of a given innovation in each attribute area. This took the form of a 0-5 rating system, where a score of 0 represents a predicted outcome of no transformative change relative to the current state of medical science and treatment, a score of 1 indicates a very insignificant transformative impact relative to current conditions in medical science and treatment, and a score of 5 indicates a very significant transformative impact relative to current conditions in medical science and treatment. In the survey, respondents are given the opportunity to include any additional attribute areas they feel are important to transformative impact in the medical space that is not present in the evaluation set provided as well as the opportunity to score the medical innovations along with those attributes*.

The results from the assessment of the early signal analysis by the Scientific Advisory Committee on which of the emerging medical innovations held the highest potential for transformative impacts set the stage for considering candidates for the Sanford Lorraine Cross Award. In doing so, we now have an award focused on the key issues of our time – celebrating transformative medical innovations of today and the researchers and clinicians playing a key role in overcoming scientific challenges, forging collaborations and ensuring that the emerging transformative medical innovation crosses the finish line to improving the lives of patients.

## 3. Conclusions

To address the need for a new type of medical innovation award that celebrates those medical researchers and innovators demonstrating the determination and success in advancing translational research for emerging transformative medical innovations, the Sanford Lorraine Cross Award has developed a unique data-driven methodology that inverts the traditional medical award selection paradigm – truly innovative areas of discovery and breakthrough science are identified independently of candidates and used to then focus candidate selection on the areas with the most promising transformative potential for patients. This unique approach allows identification of medical innovations on the cusp of achieving breakthrough outcomes for patients and allows the award to target individuals leading those emerging transformative development efforts.

For the inaugural award process, a robust set of different medical innovation areas were identified that are actively contributing to cutting edge science across a variety of medical disciplines. The area of emerging transformative medical innovation ultimately selected for the inaugural award from this grouping that had the highest potential for near term breakthroughs was centered around gene therapy applications. Alongside key advancements in the development of modified viral delivery vectors such as tailored adeno-associated viruses, recent activity in this space has addressed issues with the delivery of fragile DNA molecules without degradation or potentially dangerous immune responses.

The pioneering innovators identified within this space leading activities that are having clinical impacts today that was considered as finalists for the Lorraine Cross award were as follows:

- *Jean Bennett, M.D., Ph.D., and Katherine A. High, M.D., whose work with the RPE65 mutation has reversed an inherited form of blindness. Bennett and High pioneered gene therapy, took it to clinical trials, and then received FDA-approval for the treatment, the first FDA approval of a gene therapy for a genetic disease. High also cofounded Spark Therapeutics, a fully integrated, commercial gene therapy company working to accelerate the timeline for bringing new gene therapies to market. Bennett is a professor of ophthalmology at the University of Pennsylvania, and high is president and head of research and development at Spark Therapeutics*.
- *Brian Kaspar, Ph.D., whose lab discovered a gene replacement therapy approach that seeks to change the course of spinal muscular atrophy (SMA) by addressing its genetic cause. SMA is a devastating disease that robs babies of basic muscle functions, like breathing and swallowing, and in its most severe form (Type 1), usually leads to death by age 2 years. An initial clinical trial using the AAV9 vector to treat SMA Type 1 demonstrated a dramatic survival benefit and rapid improvement in motor milestones. Kaspar is the scientific founder and chief scientific officer of AveXis, a gene therapy company that was acquired by Novartis in 2018*.
- *James M. Wilson, M.D., Ph.D., whose work helped define the scientific and ethical standards for advancing gene therapies through FDA-approved clinical trials. He is the director of the Gene Therapy Program, the Rose H. Weiss Professor and Director of the Orphan Disease Center, and a professor of Medicine and Pediatrics in the Perelman School of Medicine at the University of Pennsylvania. In 2008, Wilson and the University of Pennsylvania cofounded REGENXBIO, Inc., a clinical-stage biotech company designing gene therapy products*.

After intensive consultation, the winner selected by the Sanford International Board of the first Sanford Lorraine Cross Award was Jean Bennett and Katherine High. The inaugural winner and other finalists exemplify the criteria outlined in the vision of the award and help to validate the methodology used to identify areas of innovation activity that is well-positioned within the translational research pipeline to have significant near-term patient impacts.

Additional areas of potentially transformative medical innovation identified by the early signals process will be monitored for ongoing developments and can be included alongside an updated set of early signals data at later times to re-evaluate transformative potential relative to future activities. In addition to allowing the most relevant areas and individuals to be selected dynamically as research focuses and the state of medical science changes, this approach helps control for any bias toward certain areas of established science over time within the selection process.
